# Nine genes abundantly expressed in the epididymis are not essential for male fecundity in mice

**DOI:** 10.1111/andr.12621

**Published:** 2019-03-29

**Authors:** T. Noda, N. Sakurai, K. Nozawa, S. Kobayashi, D. J. Devlin, M. M. Matzuk, M. Ikawa

**Affiliations:** ^1^ Research Institute for Microbial Diseases Osaka University Suita Osaka Japan; ^2^ Center for Drug Discovery Baylor College of Medicine Houston TX USA; ^3^ Department of Pathology & Immunology Baylor College of Medicine Houston TX USA; ^4^ Graduate School of Pharmaceutical Sciences Osaka University Suita Osaka Japan; ^5^ Interdepartmental Program in Translational Biology & Molecular Medicine Baylor College of Medicine Houston TX USA; ^6^ Department of Molecular and Human Genetics Baylor College of Medicine Houston TX USA; ^7^ Department of Molecular and Cellular Biology Baylor College of Medicine Houston TX USA; ^8^ Department of Pharmacology and Chemical Biology Baylor College of Medicine Houston TX USA; ^9^ Institute of Medical Science The University of Tokyo Tokyo Japan

**Keywords:** genetically modified mice, genome editing, sperm maturation

## Abstract

**Background:**

Spermatozoa become competent for fertilization during transit through the epididymis. As spermatozoa from the proximal caudal epididymis can fertilize eggs, proteins from the caput and corpus epididymis are required for sperm maturation.

**Objectives:**

Microarray analysis identified that more than 17,000 genes are expressed in the epididymis; however, few of these genes demonstrate expression restricted to the epididymis. To analyze epididymis‐enriched gene function in vivo, we generated knockout (KO) mutations in nine genes that are abundantly expressed in the caput and corpus region of the epididymis.

**Materials and methods:**

KO mice were generated using the CRISPR/Cas9 system. The histology of the epididymis was observed with hematoxylin and eosin staining. KO males were caged with wild‐type females for 3–6 months to check fertility.

**Results:**

We generated individual mutant mouse lines having indel mutations in *Pate1*,* Pate2*, or *Pate3*. We also deleted the coding regions of *Clpsl2, Epp13*, and *Rnase13*, independently. Finally, the 150 kb region encoding *Gm1110*,* Glb1l2*, and *Glb1l3* was deleted to generate a triple KO mouse line. Histology of the epididymis and sperm morphology of all KO lines were comparable to control males. The females mated with these KO males delivered pups at comparable numbers as control males.

**Discussion and conclusion:**

We revealed that nine genes abundantly expressed in the caput and corpus epididymis are dispensable for sperm function and male fecundity. CRISPR/Cas9‐mediated KO mice generation accelerates the screening of epididymis‐enriched genes for potential functions in reproduction.

## Introduction

Spermatozoa released from the testis are incapable of fertilizing eggs until they acquire capabilities necessary for fertilization competence (such as motility, capacitation, acrosome reaction, and sperm–egg fusion capabilities) by translocating through the epididymis. This step is called ‘sperm maturation’ (Robaire & Hermo, 1988; Robaire *et al*., 2006). The epididymis is mainly composed of three regions: the head (caput), body (corpus), and tail (cauda). In many mammalian species, the sperm journey from the caput region to the cauda region takes about 10 days (Robaire & Hermo, 1988). The timing of acquisition of fertilization competence by spermatozoa varies among mammalian species; however, almost all spermatozoa isolated from the proximal cauda epididymis have fertilizing ability (Robaire & Hermo, 1988), suggesting that factors from the caput and corpus epididymis play important roles in sperm maturation.

Microarray analysis shows that more than 17,000 genes are expressed in the epididymis with gene expression patterns varying greatly among regions of the epididymis (Johnston *et al*., 2005), suggesting that epididymis‐specific or epididymis‐enriched genes play important roles during sperm maturation. However, the physiological function of many of these genes remains unknown.

The emergence of the clustered regularly interspaced short palindromic repeats (CRISPR)/Cas9 system opened a new era in mammalian genome editing (Cong *et al*., 2013; Wang *et al*., 2013; Yang *et al*., 2013). The guide RNA (gRNA)/Cas9 complex recognizes 20 nucleotides upstream of the protospacer adjacent motif (PAM) as the target sequences, and then, the Cas9 nuclease causes a blunt end double‐strand break (DSB) between the 3rd and 4th nucleotides upstream of the PAM. After DSB formation, non‐homologous end‐joining (NHEJ) causes an indel mutation. We previously reported that indel mutations were efficiently obtained by injecting single gRNA and Cas9‐expressing plasmids into eggs (Mashiko *et al*., 2013). In conjunction with conventional ES cell‐mediated gene targeting, we also demonstrated that many testis‐enriched genes are not essential for male fertility (Miyata *et al*., 2016). Our previous work demonstrated that CRISPR/Cas9‐mediated KO mice generation, and phenotype screening is a cost‐effective and labor‐effective approach to quickly identify essential gene functions in vivo (Mashiko *et al*., 2014).

In the present study, we have knocked out nine epididymis‐enriched genes and discovered these genes are not essential for sperm fertilizing ability and male fecundity.

## Materials and Methods

### Animals

All mice used in this study were purchased from Japan SLC (Hamamatsu, Shizuoka, Japan) or CLEA Tokyo, Japan. Mice were acclimated to a 12‐h light/12‐h dark cycle. All animal experiments were approved by the Animal Care and Use Committee of the Research Institute for Microbial Diseases, Osaka University, Japan (#Biken‐AP‐H30‐01) and the Institutional Animal Care and Use Committee of Baylor College of Medicine (AN‐716).

### cDNA and RT‐PCR

All tissues [brain, heart, kidney, liver, lung, spleen, thymus, ovary, uterus, testis, epididymis (caput, corpus, and cauda regions), prostate (mixture of dorsal, lateral, and ventral regions), coagulating gland, and seminal vesicle] were collected from C57BL/6NCr mice. These samples were homogenized in TRIzol (Ambion, Foster City, CA, USA). The total RNA was reverse‐transcribed to cDNA using SuperScript III First Strand Synthesis System for RT‐PCR (Invitrogen, Carlsbad, CA, USA). Five ng of cDNA was used for PCR with primer sets (Table S1) and KOD DNA Polymerase (KOD‐FX Neo, Toyobo, Osaka, Osaka, Japa).

### Gene tree

The gene tree was made by GENETYX with the amino acid sequence (method: UPGMA) [for Colipase like 2 (CLPSL2): NP_001030043 (mouse), Q6UWE3 (human), XP_003311311 (chimpanzee), XP_022281476 (dog)] [for Epididymal protein 13 (EPP13): NP_001170887 (mouse), NP_001341587 (human), PNI23622 (chimpanzee), XP_024835189 (cow)] [for GM1110: XP_006510522 (mouse), ENSCAFT00000043177 (dog), ENSBTAT00000016226 (cow)] [for Galactosidase, beta 1‐like (GLB1L) 2: XP_006510360 (mouse), NP_612351 (human), XP_009422813 (chimpanzee), XP_022273772 (dog), XP_010811276 (cow)] [for GLB1L3: AAI32203 (mouse), AAH11001 (human), XP_024203264 (chimpanzee), XP_005620347 (dog), XP_024831720 (cow)] [for Prostate and testis expression (PATE) 1: NP_001186882 (mouse), NP_612151 (human), XP_024203322 (chimpanzee), XP_022273825 (dog), XP_024843340 (cow)] [for PATE2: XP_011240865 (mouse), NP_997720 (human), XP_001148077 (chimpanzee), XP_005619644 (dog), XP_024843404 (cow)] [for PATE3: NP_001161064 (mouse), NP_001123355 (human), XP_024203289 (chimpanzee), XP_022273771 (dog), XP_024843215 (cow)] [for RNase A family 13 (RNASE13): XP_017171565 (mouse), AAV87186 (human), XP_003804788 (chimpanzee), XP_013974975 (dog), AAI34657 (cow)].

### Egg collection

Pregnant mare serum gonadotropin (PMSG) (5 units, ASKA Pharmaceutical, Tokyo, Japan) or CARD HyperOva (0.1 mL, Kyudo, Tosu, Saga, Japan) was injected into the abdominal cavity of B6D2F1 females, followed by human chorionic gonadotropin (hCG) (five units, ASKA Pharmaceutical) and natural mating with B6D2F1 males 48 h after PMSG or CARD HyperOva. After 20 h, we collected fertilized eggs with two pronuclei for genome editing.

### Pronuclear injection

Pronuclear injection with 5 ng/μL of gRNA/Cas9‐expressing plasmid (for *Pate1*,* Pate2,* and *Pate3*) was performed as previously reported (Mashiko *et al*., 2013; Noda *et al*., 2017). The crRNA and tracrRNA (Sigma‐Aldrich, St. Louis, MO, USA) were diluted with nuclease‐free water (non‐DEPC treated, Ambion). The mixture was denatured at 95 °C for 1 min and allowed to anneal by cooling gradually to room temperature (~1 h). Each gRNA was mixed with Cas9 protein solution (Thermo Fisher Scientific, Waltham, MA, USA) and T_10_E_0.1_ buffer (10 mM Tris‐HCl, 0.1 mM EDTA, pH 7.4), and then incubated at 37 °C for 5 min to prepare the gRNA/Cas9 RNPs. For multiple gene targeting, we prepared the gRNA/Cas9 RNP solution separately and then combined them [final concentration: 30 ng/μL (≈ 200 nM) Cas9 for 20 ng/μL (≈ 600 nM) of each gRNA (Table S2)]. After centrifugation at 20,000 g at 4 °C for 10 min, the mixture was used for pronuclear injection (for *Clpsl2*,* Epp13*,* Rnase13*,* Gm1110*,* Glb1l2*, and *Glb1l3*).

### Electroporation

The gRNA was prepared as described above. The gRNA was mixed with Cas9 protein solution and opti‐MEM media (Thermo Fisher Scientific), and then incubated at 37 °C for 5 min to prepare the gRNA/Cas9 RNPs [final concentration: 50 ng/μL (≈ 330 nM) Cas9 for 20 ng/μL (≈ 600 nM) of each gRNA (Table S2)]. The gRNA/Cas9 RNP solution was placed between the electrodes with a 5 mm gap in the NEPA21 Super Electroporator (Nepagene, Ichikawa, Chiba, Japan). Fertilized eggs were arranged between the electrodes, and then, the electroporation was done with the following conditions [resistance value: 550~600 Ω, poring pulse: 225 V (voltage), 2 ms (pulse amplitude), 50 ms (pulse interval), four (pulse number), 10% (attenuation), + (polarity), transfer pulse: 20 V (voltage), 50 ms (pulse amplitude), 50 ms (pulse interval), ± 5 (pulse number), 40% (attenuation), +/− (polarity)].

### Egg transfer

Injected and electroporated embryos were transplanted into the uterus of pseudo‐pregnant ICR recipients. After 19 days, offspring were obtained by natural birth or Caesarean section.

### Epididymis histology and sperm morphology

Epididymides were fixed in Bouin's fluid (Polysciences, Warrington, PA, USA) at 4 °C overnight. Fixed epididymides were dehydrated by increasing ethanol concentrations and then were embedded with paraffin. Paraffin sections (5‐μm) were stained with Mayer hematoxylin solution for 3 to 5 min, counterstained with eosin Y solution [53% (v/v) ethanol, 0.3% (v/v) eosin, and 0.5% (v/v) acetic acid] for 2 to 5 min, dehydrated in increasing ethanol concentrations, and finally mounted in Permount or Entellan new (Merck, Kenilworth, NJ, USA). The caudal epididymal spermatozoa were observed with phase contrast microscopy.

### Mating test

KO male mice were caged with 2 or 3 B6D2F1 females (for *Pate1*,* Pate2*,* Pate3*,* Epp13*,* Rnase13*, and *Glb1l2*‐*Gm1110* mutant mice), or 1 hybrid female (C57BL/6J × 129S5/SvEvBrd.) (for *Clpsl2*) for 3 to 6 months. Frozen spermatozoa from *Pate1*‐, *Pate2*‐, *Pate3*‐, *Clpsl2*‐disrupted males (B6D2‐*Pate1 *<* *em1Osb>, RBRC#09830, CARD#2450; B6D2‐*Pate2 *<* *em1Osb>, RBRC#09831, CARD#2451; B6D2‐*Pate3 *<* *em1Osb> *Pate3 *<* *em2Osb>, RBRC#09832, CARD#2452; B6D2‐*Clpsl2 *<* *em1Osb>, RBRC#10341, CARD#2711) will be available through RIKEN BRC (http://en.brc.riken.jp/index.shtml) and CARD R‐BASE (http://cardb.cc.kumamoto-u.ac.jp/transgenic/).

### Statistical analysis

All values are shown as the mean ± SD of at least three independent experiments. Statistical analyses were performed using Student's *t*‐test (Figs 2, 3, 4 and 6) and Mann–Whitney test (Fig. 5).

## Results

### Selection of the target genes

**Figure 1:**
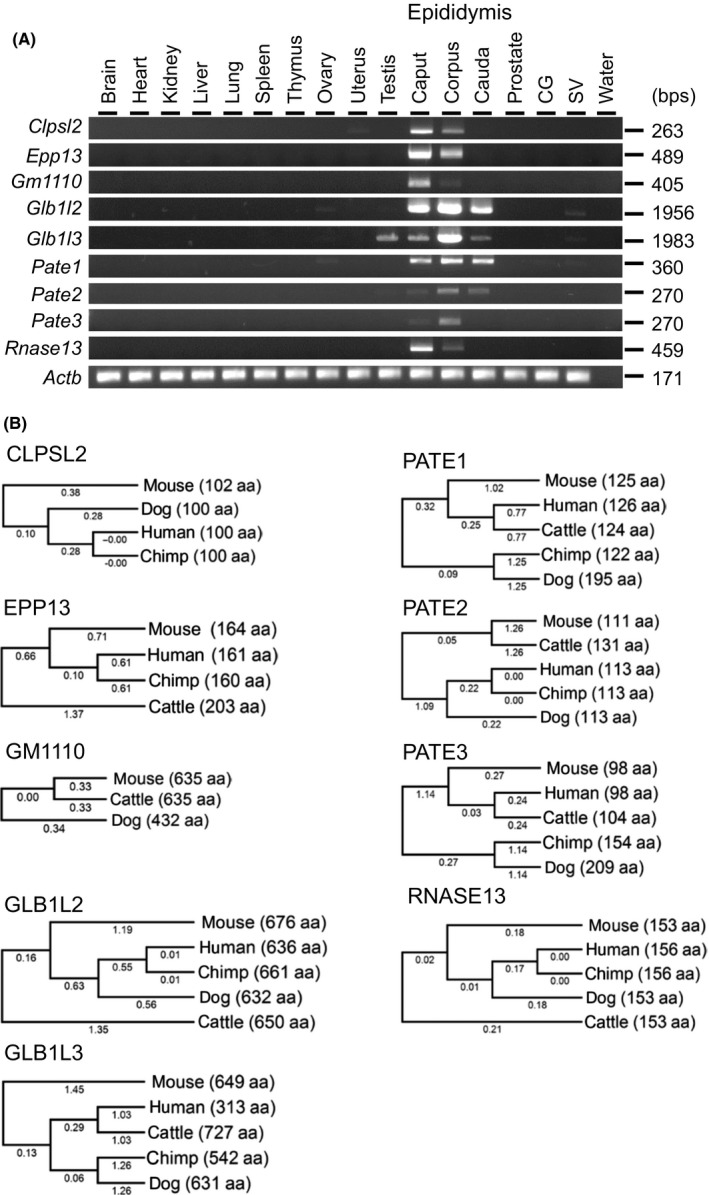
Tissue expression analysis and conservation of target genes between some mammalian species. (A) RT‐PCR analysis. Actin beta (*Actb*) was used as the control CG: coagulating gland, SV: seminal vesicle. (B) Phylogenetic trees. The values under branches and parentheses show the distances and the lengths of the amino acid sequences, respectively.

We selected nine genes abundantly expressed in the epididymis and reconfirmed their expression by RT‐PCR (Fig. 1A). While *Glb1l3* showed detectable expression in testis, the remaining genes showed epididymis‐enriched expression. *Clpsl2*,* Epp13*,* Gm1110*, and *Rnase13* were strongly expressed in the caput region, while *Glb1l2*,* Glb1l3*,* Pate2*, and *Pate3* were strongly expressed in the corpus region, and *Pate1* was expressed in all regions at comparable levels. It should be noted that all genes examined in the study, except *Gm1110*, are conserved in human and various species (Fig. 1B). Because *Gm1110*,* Glb1l2*, and *Glb1l3* located closely on the same chromosome and show high similarity in amino acid sequence (Figure S1), we decided to generate a null mice without these three genes.

### Fertility of *Pate* family knockout mice

**Figure 2:**
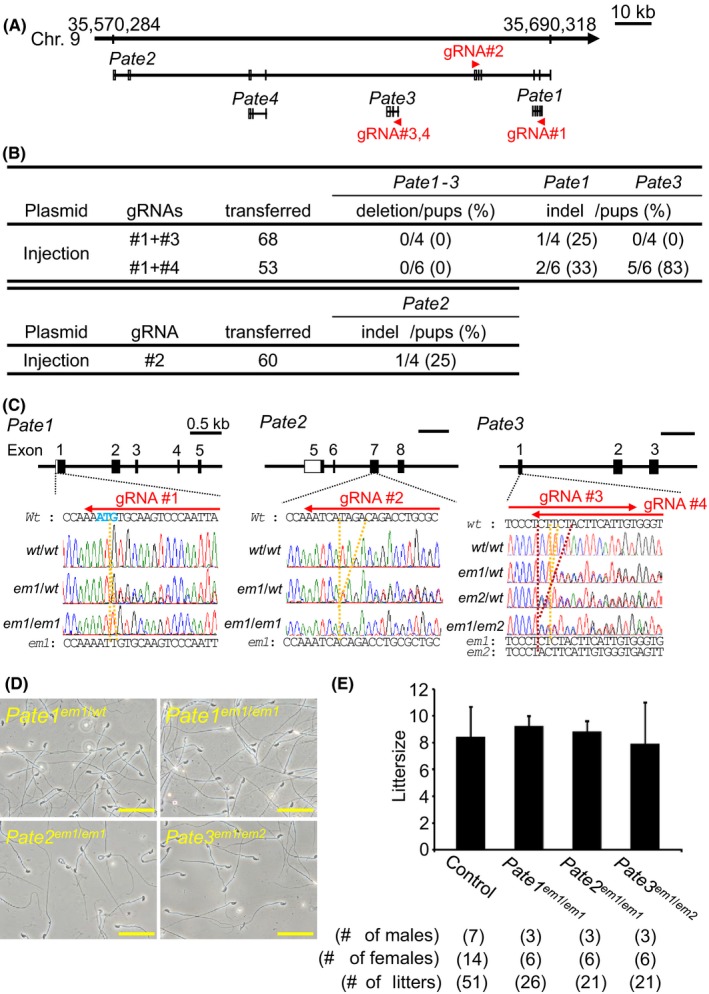
Fecundity of KO males of *Pate* family genes. (A) *Pate* family genes within murine genomic locus |chromosome 9qA4|. (B) Genome editing efficiency of injecting gRNA/Cas9‐expressing plasmids into eggs. (C) DNA sequencing of KO mice of *Pate* family genes. Enzyme mutation (*em*) 1 for *Pate1*: 1 base ‘T’ insertion; *em1* for *Pate2*: 4 base ‘TAGA’ deletion; *em1* and *em2* for *Pate3*: 1 base ‘T’ and 5 base ‘CTTCT’ deletion. Black boxes: coding region, Blue colored letters: initial methionine. (D) Sperm morphology observed under phase contrast. (E) Male fecundity. There was no difference in average litter size between control and KO males of each gene (*Pate1*:* p* = 0.57, *Pate2*:* p* = 0.80, *Pate3*:* p* = 0.76). Heterozygous males of each gene were used as controls. [Colour figure can be viewed at wileyonlinelibrary.com]

Four and thirteen *Pate* family genes form a cluster on human chromosome 11 and mouse chromosome 9 (Levitin *et al*., 2008), respectively. While *Pate4* is abundantly expressed in mouse seminal vesicles and not expressed in the epididymis (Noda *et al*., 2018), *Pate1*,* 2*, and *3* are exclusively expressed in the epididymis (Fig. 1A). Because we reported that *Pate4* KO mice are subfertile due to an impaired vaginal plug formation and semen leakage (Noda *et al*., 2018), we knocked out the remaining members to determine their roles in fertility. We attempted to delete the region covering all *Pate1*,* Pate2*, and *Pate3*, by injecting sgRNA/Cas9 co‐expressing plasmids into zygotes (Fig. 2A). Although we could not delete the region [deletion/pups = 0/4 (sgRNA #1 + #3), 0/6 (sgRNA #1 + #4), Fig. 2B], we obtained F0 indel mutant mice in *Pate1* and *Pate3* independently [*Pate1*: indel/pups = 1/4 (sgRNA #1 + #3), 2/6 (sgRNA #1 + #4), *Pate3*: indel/pups = 0/4 (sgRNA #1 + #3), indel/pups = 5/6 (sgRNA #1 + #4), Fig. 2B]. We additionally generated *Pate2 *F0 mutant mice [*Pate2*: indel/pups = 1/4 (sgRNA #2), Fig. 2B]. Because small indels are difficult to detect by PCR and subsequent agarose gel electrophoresis (1 bp insertion in *Pate1*, 4 bp deletion in *Pate2*, 1 and 5 bp deletion in *Pate3*), we genotyped mutant mice by sequencing PCR amplicons (Fig. 2C). KO mice were obtained by heterozygous F1 intercrosses. All the KO mice produced spermatozoa with normal morphology as shown in Fig. 2D. When we examined three males for each KO lines, all KO males showed normal fecundity [littersize: 9.3 ± 0.7 for *Pate1* ^*em1*/*em1*^, 8.8 ± 0.8 for *Pate2* ^*em1*/*em1*^, and 7.9 ± 3.1 for *Pate3* ^*em1*/*em2*^, while control showed 8.5 ± 2.2 (Fig. 2E)]. These results show that *Pate1*,* 2*, and *3* are dispensable for the male fecundity.

### Fecundity of colipase like 2 (*Clpsl2*) knockout mice

**Figure 3:**
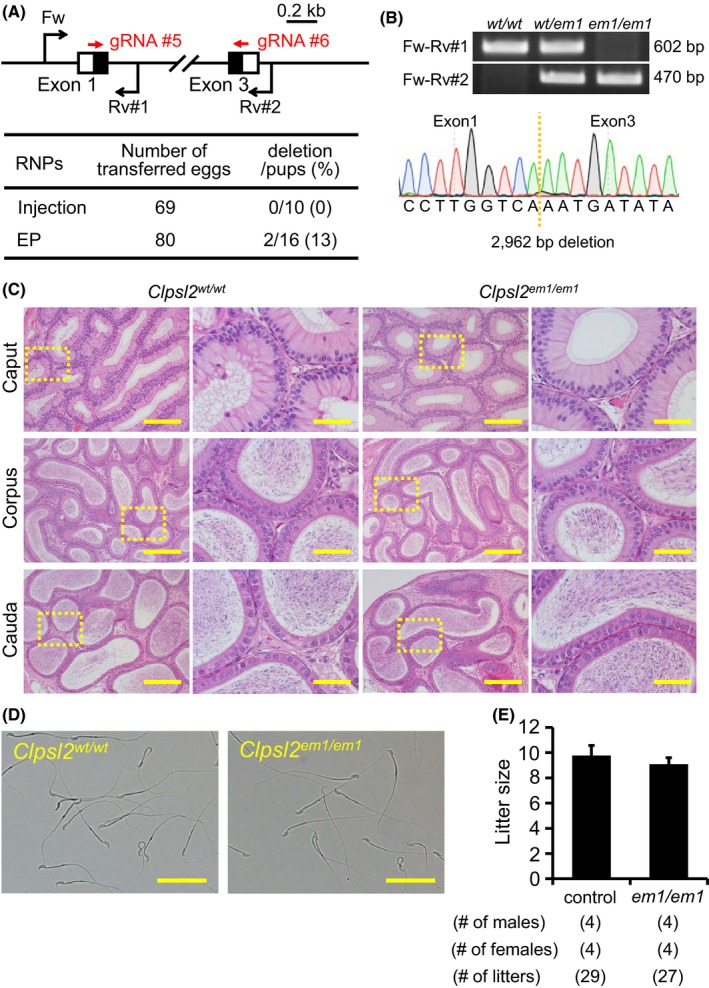
Fecundity of *Clpsl2* KO males. (A) Genome editing efficiency with gRNA/Cas9 RNPs. Black arrows: primers for genotyping, Black boxes: coding region, EP: electroporation. (B) Genotyping with PCR and DNA sequencing. Three primers (Fw, Rv#1, and Rv#2) were used for the PCR (also see panel A). *em1*: 2962 bp deletion. (C) Histological analysis with H&E staining. Dashed areas in left panels were enlarged (right panels). Scale bars on the left and right panels are 200 μm and 50 μm, respectively. (D) Sperm morphology observed under phase contrast. Scale bars: 50 μm. (E) Male fecundity. There was no difference in average litter size between WT and KO males (*p* = 0.20). [Colour figure can be viewed at wileyonlinelibrary.com]

CLPSL2 is conserved between mice and humans. Using RNAi methods, a previous paper suggested that *Clpsl2* is required for sperm maturation (Lu *et al*., 2018). To evaluate these findings and determine the essential roles of CLPSL2 in the complete absence of the protein, we generated KO mice lacking the gene. Hereafter, we applied both sgRNA/Cas9 co‐expressing plasmids and gRNA/Cas9 ribonucleoprotein (RNP) because the genome editing efficiency is comparable in various cell lines (Liang *et al*., 2015). We also applied a zygote electroporation system because many eggs can be treated in a shorter time. When we injected two gRNA/Cas9 RNPs into zygotes, we could not obtain any F0 mutant mice (deletion/pups = 0/10); however, by electroporation, we could obtain F0 deletion mutants (deletion/pups = 2/16) (Fig. 3A). The deletion of the coding region was confirmed by sequencing the PCR amplicons (Fig. 3B). KO mice were obtained by heterozygous F1 intercrosses. Histological analysis of the epididymis and analysis of sperm morphology failed to reveal any apparent differences between *Clpsl2* ^*em1*/*em1*^ and control males (Fig. 3C,D). The *Clpsl2* ^*em1*/*em1*^ males showed normal fecundity (litter size: 9.8 ± 0.8 for controls, 9.1 ± 0.5 for *Clpsl2* ^*em1*/*em1*^; Fig. 3E). Thus, *Clpsl2* is not required for male fecundity.

### Fertility of *Epp13* knockout mice

**Figure 4:**
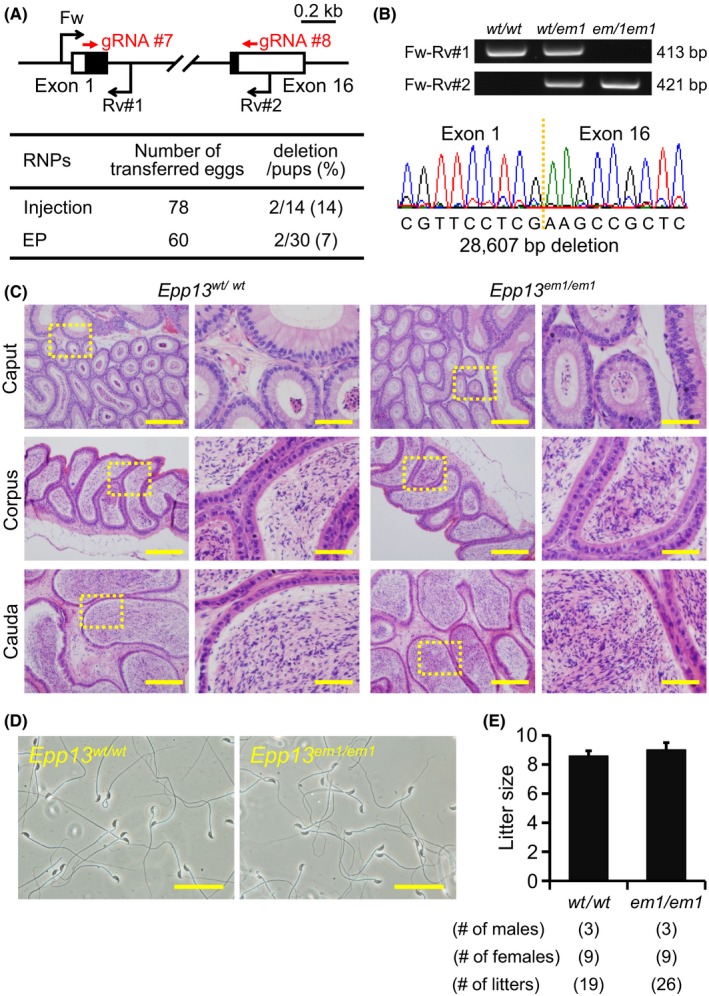
Fecundity of *Epp13* KO males. (A) Genome editing efficiency with gRNA/Cas9 RNPs. Black arrows: primers for genotyping, Black boxes: coding region, (B) Genotyping with PCR and DNA sequencing. Three primers (Fw, Rv#1, and Rv#2) were used for the PCR (also see panel A). *em1*: 28,607 bp deletion. (C) Histological analysis with H&E staining. Dashed areas in left panels were enlarged (right panels). Scale bars on the left and right panels are 200 μm and 50 μm, respectively. (D) Sperm morphology observed under phase contrast. Scale bars: 50 μm. (E) Male fecundity. There was no difference in average litter size between WT and KO males (*p* = 0.28). [Colour figure can be viewed at wileyonlinelibrary.com]

*Epp13* is conserved between mice and humans (also known as *EDDM13*). We generated *Epp13* F0 mutant mice with pronuclear injection and electroporation of gRNA/Cas9 RNPs (injection: deletion/pups = 2/14, electroporation: deletion/pups = 2/30) (Fig. 4A). The complete deletion of the coding region was confirmed by PCR and direct sequencing (Fig. 4A,B). KO mice were obtained by heterozygous F1 intercrosses. There were no apparent differences between *Epp13* ^*em1*/*em1*^ and control males in histology of epididymis and sperm morphology (Fig. 4C,D). The *Epp13* ^*em1*/*em1*^ males showed normal fecundity (litter size: 8.6 ± 0.3 for controls, 9.0 ± 0.5 for *Epp13* ^*em1*/*em1*^; Fig. 4E). Thus, *Epp13* is not required for male fecundity.

### Fertility of *Rnase13* knockout mice

**Figure 5:**
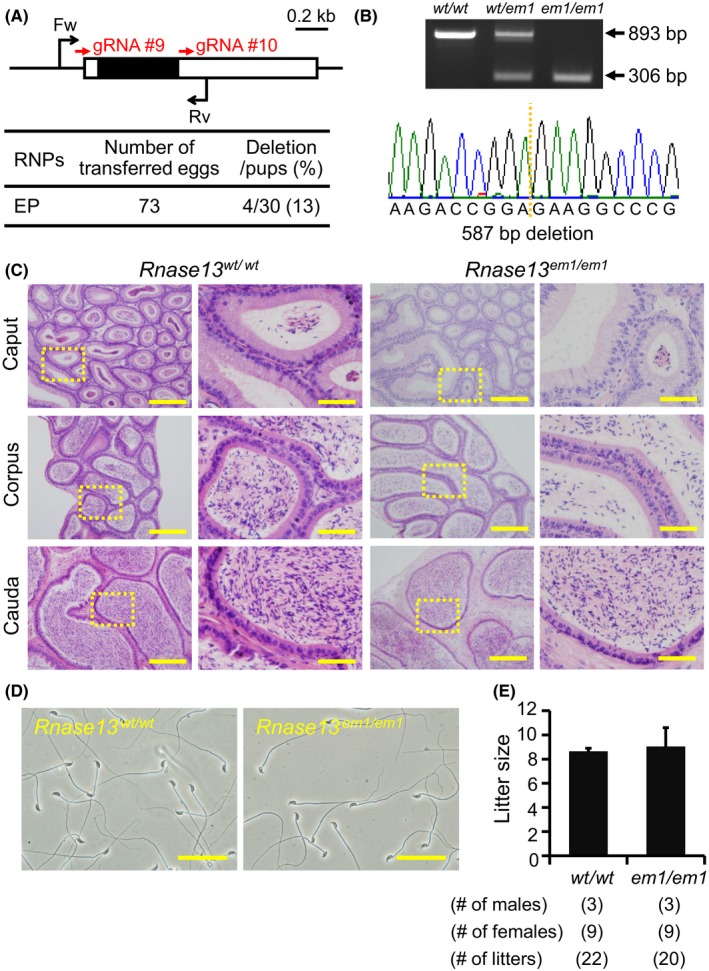
Fecundity of *Rnase13* KO males. (A) Genome editing efficiency with gRNA/Cas9 RNPs. Black arrows: primers for genotyping, Black boxes: coding region. (B) Genotyping with PCR and DNA sequencing. Two primers were used for PCR (also see panel A). *em1*: 587 bp deletion, (C) Histological analysis with H&E staining. Dashed areas in left panels were enlarged (right panels). Scale bars on the left and right panels are 200 μm and 50 μm, respectively. (D) Sperm morphology observed under phase contrast. Scale bars: 50 μm. (E) Male fecundity. There was no difference in average litter size between WT and KO males (*p* = 0.51). [Colour figure can be viewed at wileyonlinelibrary.com]

*Rnase13*, conserved between mice and humans, is a member of the ribonuclease A superfamily members (Cho *et al*., 2005), of which some members, *Rnase9* and *Rnase10*, have been shown to be required for sperm maturation (Krutskikh *et al*., 2012; Westmuckett *et al*., 2014). We generated *Rnase13* F0 mutant mice with 2 gRNAs (deletion/pups = 4/30) (Fig. 5A). The deletion of the coding region was confirmed by PCR and direct sequencing (Fig. 5B). KO mice were obtained by heterozygous F1 intercrosses. There were no apparent differences between *Rnase13* ^*em1*/*em1*^ and control males in histology of epididymis and sperm morphology (Fig. 5C,D). The *Rnase13* ^*em1*/*em1*^ males showed normal fecundity (litter size: 8.6 ± 0.2 for controls, 9.0 ± 1.6 for *Rnase13* ^*em1*/*em1*^, Fig. 5E). Thus, *Rnase13* is not required for male fecundity.

### Fertility of mice lacking *Glb1l2*,* Glb1l3*, and *Gm1110*

**Figure 6:**
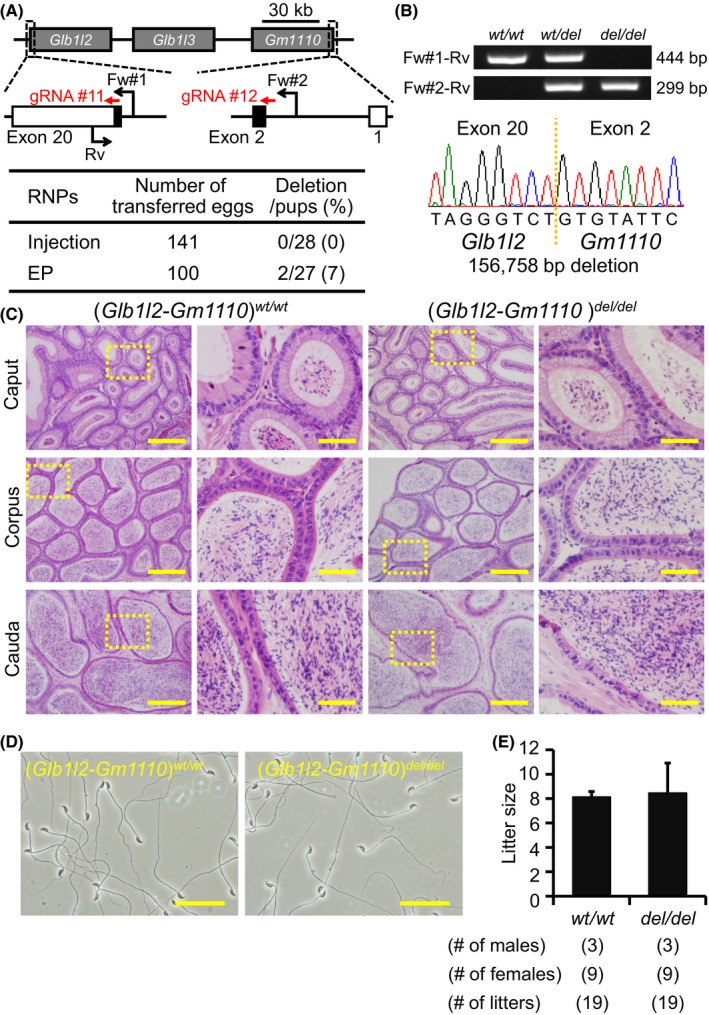
Fecundity of *Glb1l2‐Gm1110* KO mice. (A) Genome editing efficiency with gRNA/Cas9 RNPs. Black arrows: primers for genotyping, Black boxes: coding region. (B) Genotyping with PCR and DNA sequencing. Three primers (Fw#1, Fw#2, and Rv) were used for the PCR (also see panel A). *em1*: 156,758 bp deletion. (C) Histological analysis with H&E staining. Dashed areas in left panels were enlarged (right panels). Scale bars on the left and right panels are 200 μm and 50 μm, respectively. (D) Sperm morphology observed under phase contrast. Scale bars: 50 μm. (E) Male fecundity. There was no difference in average litter size between WT and KO males (*p* = 0.83). [Colour figure can be viewed at wileyonlinelibrary.com]

*Glb1l2 and Glb1l3* are categorized as galactosidases. *Gm1110* is located next to *Glb1l3* on mouse chromosome 9 (Fig. 6A) (Zhen *et al*., 2009). The homology rates between GLB1L2, GLB1L3, and GM1110 are high at the amino acid level (Figure S1, Gm1110 vs. GLB1L2: 47.4%, Gm1110 vs. GLB1L3: 44.9%), suggesting redundant functions of these genes on sperm maturation. *Gm1110* is identified as *Glb1l4* in rats, and a previous report suggested that GLB1L4 is required for the sperm maturation (Zhen *et al*., 2009). Thus, we generated 2 gRNAs against *Glb1l2* and *Gm1110* and deleted all 3 genes (injection: deletion/pups = 0/28, electroporation: deletion/pups = 2/27) (Fig. 6A). The deletion was confirmed by PCR and direct sequencing (Fig. 6B). KO mice were obtained by heterozygous F1 intercrosses. There were no apparent differences between triple KO (*Glb1l2‐Gm1110*)^*del*/*del*^ and control males in histology of epididymis and sperm morphology (Fig. 6C,D). The triple KO males showed normal fecundity [litter size: 8.2 ± 0.4 for controls, 8.5 ± 2.4 for (*Glb1l2‐Gm1110*)^*del*/*del*^, Fig. 6E]. Thus, *Glb1l2, Glb1l3,* and *Gm1110* are not required for male fecundity.

## Discussion

It is known that 4 and 13 genes of the *Pate* family members exist in human and mouse, respectively, and that *Pate1* to *Pate4* are conserved in both species (Levitin *et al*., 2008). In human, PATE1 was localized to the sperm equatorial segment, and the anti‐PATE1 antibody treatment decreased sperm motility (Liu *et al*., 2015). Further, previous studies showed asthenozoospermia patients having reduced levels of PATE1 (Liu *et al*., 2015), and that single nucleotide polymorphisms were found in idiopathic asthenozoospermia (Zhang *et al*., 2016). These results suggest that human PATE1 is required for sperm function, but we revealed that mouse *Pate1* KO males are fertile (Fig. 2D,E). In addition, we showed that mouse *Pate2* and *Pate3* are dispensable for male fecundity. Because more *Pate* family genes exist in mouse, we cannot rule out the possibility of gene complementation. Further studies will be required to fully uncover the role of PATE family protein in sperm functions.

*Clpsl2* is conserved in mouse and human, and predominantly expressed in the caput epididymis (Fig. 1). CLPSL2 from the caput epididymis binds to the sperm acrosome and principal piece of the sperm tail (Lu *et al*., 2018). The previous study showed that knockdown of mouse *Clpsl2* by in vivo lentivirus‐based RNAi resulted in decreased sperm motility, acrosome reaction, and sperm count in the cauda epididymis, leading to the male subfertility (Lu *et al*., 2018). However, we could not observe any defects in *Clpsl2* KO (null) males. The discrepancy may be explained by experimental approaches; while KO mice specifically suppressed the target gene, RNAi interferes not only with the target gene, but also genes carrying similar sequences. While one can claim that the gRNA may also have off‐target effects, our data support that *Clpsl2* is not essential for male fertility.

RNASE9, 10, and 13 in the ribonuclease A superfamily were detected in the epididymis (Penttinen *et al*., 2003; Westmuckett *et al*., 2014) and show high homology rates at the amino acid level (Cho *et al*., 2005). *Rnase9* KO males were fertile, but KO spermatozoa showed impaired sperm maturation (Westmuckett *et al*., 2014). *Rnase10* KO males were severely subfertile due to the disruption of ADAM3 processing (Krutskikh *et al*., 2012). However, we revealed that *Rnase13* is dispensable for male fecundity. We cannot rule out the possibility that other members complement RNASE13 function in vivo, so the additional KO approaches will be necessary to address this question. Because RNase A family genes are located on Chromosome 14, two gRNAs mediated deletion approaches as shown in Fig. 6 are feasible.

Here, we showed that nine genes, which are abundantly expressed in epididymis and conserved among several mammalian species, are dispensable for sperm maturation and male fecundity. The CRISPR/Cas9‐mediated KO approach is a powerful tool to determine whether genes of interests are essential for fertility. Screening for genes through a CRISPR/Cas9‐mediated KO approach has accelerated the study of severed genes implicated in reproduction, especially of genes within gene families.

## Funding Information

This work was supported by the Ministry of Education, Culture, Sports, Science, and Technology (MEXT)/Japan Society for the Promotion of Science (JSPS) KAKENHI grants (JP18K14612 to T.N., JP17H01394 and JP25112007 to M.I.), Japan Agency for Medical Research and Development (AMED) grant (JP18gm5010001), Takeda Science Foundation grants to M.I., the Eunice Kennedy Shriver National Institute of Child Health and Human Development (P01HD087157 and R01HD088412 to M.M.M. and M.I.), the Bill & Melinda Gates Foundation (Grand Challenges Explorations grant OPP1160866 to M.M.M. and M.I.), a Japan Society for the Promotion of Science Overseas Research Fellowships (20170633) to K.N., and a Training Interdisciplinary Pharmacology Scientists (TIPS) Program (T32 GM120011) predoctoral fellowship position to D.J.D.

## Conflict of Interest

The authors declare no conflict of interest.

## Authors’ Contributions

Designed the research: T.N., N.S., K.N., M.M.M., and M.I.; Performed the research: T.N., N.S., K.N, S.K., and D.J.D.; Analyzed the data: T.N., N.S., and K.N.; and Wrote the paper: T.N., N.S., K.N., M.M.M. and M.I.

## Supporting information


**Figure S1** Multiple sequence alignment with GM1110, GLB1L2, and GLB1L3.

**Table S1** Primer sequences for the tissue expression analysis.

**Table S2** Primer sequences for the genotyping and gRNA sequences.
