# Supplementary material for: Nine genes abundantly expressed in the epididymis are not essential for male fecundity in mice
Source: Andrology. 2019 Mar 29;7(5):644–53. doi: 10.1111/andr.12621 (PMC6688925; doi:10.1111/andr.12621)
Supplement: Supplementary file 1 — Figure S1 Multiple sequence alignment with GM1110, GLB1L2, and GLB1L3. [file ANDR-7-644-s001.pdf]

Supplemental Fig. S1 (Noda et al.)

```
mGM1110/1-635     1 --MWAFLGRPE----------------------------------CILCLSSAL--CAMA  22
mGLB1L2/1-676     1 MPKWSLRRRPGRTLGLLLLLVLSFLVLRSPRVFMLNVVSVLFLAAASACSSPAVGPSPGS  60
mGLB1L3/1-649     1 -------------------------------------MAIFFLPTV------LSGFAPHS  17

mGM1110/1-635    23 LLSNNEFSRLNQTHLTPLLLINRKVGLQVKD-----SQFTLEGFPFRIISGTIDYFRIPR  77
mGLB1L2/1-676    61 SAEESHLSRLNWSTLVPLWLQHQRLGLRTKG-----PDFILEDSIFQILGGSIHYFRVPR 115
mGLB1L3/1-649    18 EEVFKSPARFSWSNLTPSELKNRFVGLSTKTNALGKAYFTLEGHKFMIVGGSIHYFRVPR  77

mGM1110/1-635    78 NSWRLSLRKMQAGGFNTLTTHIPWNLHEPAVGQFYFTENTDLIAFITMASQSGLWVILCP 137
mGLB1L2/1-676   116 EYWRDRLLKLKACGLNTLTTYVPWNLHEPERGKFDFSGNLDLEAFIQLAAKIGLWVILRP 175
mGLB1L3/1-649    78 EYWKDRLLKLQACGFNTVTTYIPWNLHEQERGKFDFSEILDLEAYVLLAKTIGLWVILRP 137

mGM1110/1-635   138 GPYIGSDLDLGGLPSWLLKDPKMKLRTTYKGFTKAMNRYFDNLIPKIAKFQYKKGGPIIA 197
mGLB1L2/1-676   176 GPYICSEIDLGGLPSWLLQDPDMKLRTTYHGFTKAVDLYFDHLMSRVVPLQYKHGGPIIA 235
mGLB1L3/1-649   138 GPYICAEVDLGGLPSWLLRNPVTDLRTTNKGFIEAVDKYFDHLIPKILPLQYRHGGPVIA 197

mGM1110/1-635   198 VQVENEYGSYYMDKKYMAYVKTALVSRGIDELLMTADDGVSLRKGHLENVLATVHMKNIK 257
mGLB1L2/1-676   236 VQVENEYGSYNKDRAYMPYIKKALEDRGIIEMLLTSDNKDGLEKGVVDGVLATINLQSQQ 295
mGLB1L3/1-649   198 VQVENEYGSFQKDRNYMNYLKKALLKRGIVELLLTSDDKDGIQIGSVNGALTTINMNSFT 257

mGM1110/1-635   258 KETF--GDLRSIQGKSPILMMVYTTKSFDTWGTLHQTGDAQMLLKDVHEMFHLGFSLNFY 315
mGLB1L2/1-676   296 ELMALNTVLLSIQGIQPKMVMEYWTGWFDSWGGSHNILDSSEVLQTVSAIIKDGSSINLY 355
mGLB1L3/1-649   258 KDSF--IKLHKMQSDKPIMIMEYWTGWYDSWGSKHIEKSAEEIRHTVYKFISYGLSFNMY 315

mGM1110/1-635   316 MFQGGTNFGLIGGAQSSEGYKPVVTSYDYNALVSEGGEYTVQYREFQRFFHSVTDSHLTV 375
mGLB1L2/1-676   356 MFHGGTNFGFINGAMHFNDYKADVTSYDYDAILTEAGDYTAKYTKLRELFGTVSGIPPPP 415
mGLB1L3/1-649   316 MFHGGTNFGFINGGRYENHHISVVTSYDYDAVLSEAGDYTEKYFKLRKLFASGSARPLPP 375

mGM1110/1-635   376 QPKTTQMFAYQPVTLLYFMTLWEFLPNLVKTTKSSKPLSMEQLRVNERSGQSFGYILYET 435
mGLB1L2/1-676   416 PPELTAKMVYEPMSPALYLSLWDAIQYMDKPVTSETPINMENLPVNNGNGQAFGYVLYET 475
mGLB1L3/1-649   376 LPPLIPKAIYPSVNLSFYLPLFDILPYLNQPVMLHTPVTMENLPINNGSGQPFGLVLYET 435

mGM1110/1-635   436 VIFSGGLITSRGHIRDRGQVFLDNIYIGLLDQSNSELLLYKDVSKKSQILRILVENQGRL 495
mGLB1L2/1-676   476 TIFSSGVLS--GLVRDRGQVFLNRVSIGFLDYKTTKITI--PLTQGYTILRILVENRGRV 531
mGLB1L3/1-649   436 SICSGGSLF--ASVHDSAQVFLNDQSIGILDENHEVLQI--PKIQGCQLLRILVENQGRI 491

mGM1110/1-635   496 TSGQDINKERKGLTGDIYLDKSPLRPFKIYSLEMNNIFIQREFPNSWQRVTSQVQGPAFF 555
mGLB1L2/1-676   532 NYGNNIDSQRKGLIGNLYLNNKALKNFKIYSLDMTKQFLQRFDMDNWSVIPKELTFPAFF 591
mGLB1L3/1-649   492 NYSWRIQSEQKGLKEVVSIDGLLLTNFTIYSLDMKMSFFKRLRSASWRLAPKTYNGPAFY 551

mGM1110/1-635   556 LSYLKAGDPPQDTFMKIQGWGKGVISINGRSLGRYWNIGPQETVFVPGSWLQPGVNKIIM 615
mGLB1L2/1-676   592 LGALSVGIYPSDTFLKLEGWVKGVVFVNGHNLGRYWNVGPQETLYLPGVWLDKGINKVII 651
mGLB1L3/1-649   552 WGILNAGSSPTDTFLHLPNWHYGFVFINGRNLGRYWDIGPQRTLYLPGPWLHPEDNEVIV 611

mGM1110/1-635   616 FEELKGDEKIHFSTKAQLGH------------------                       635
mGLB1L2/1-676   652 FEETMSGSMVQSTDIPHLGRNQYIN-------------                       676
mGLB1L3/1-649   612 FEKIEKGFDIQTRKKPQLQECYETGSGLPDAAGDADPC                       649
```
